# Recycling of Sustainable Co-Firing Fly Ashes as an Alkali Activator for GGBS in Blended Cements

**DOI:** 10.3390/ma8020784

**Published:** 2015-02-16

**Authors:** Yann-Hwang Wu, Ran Huang, Chia-Jung Tsai, Wei-Ting Lin

**Affiliations:** 1 Institute of Materials Engineering, National Taiwan Ocean University, 2 Pei-Ning Road, Keelung 20224, Taiwan; E-Mail: 20055002@mail.ntou.edu.tw; 2 Taiwan & Pacific Engineers & Constructors Ltd., Taipei 10669, Taiwan; 3 Department of Harbor and River Engineering, National Taiwan Ocean University, 2 Pei-Ning Road, Keelung 20224, Taiwan; E-Mail: ranhuang@ntou.edu.tw; 4 Department of Civil Engineering, National Ilan University, I-Lan 26047, Taiwan; E-Mail: wtlin@niu.edu.tw

**Keywords:** sustainable materials, co-firing fly ashes, GGBS, innovative alkali activators

## Abstract

This study investigates the feasibility of co-firing fly ashes from different boilers, circulating fluidized beds (CFB) or stokers as a sustainable material in alkali activators for ground granulated blast-furnace slag (GGBS). The mixture ratio of GGBS and co-firing fly ashes is 1:1 by weight. The results indicate that only CF fly ash of CFB boilers can effectively stimulate the potential characteristics of GGBS and provide strength as an alkali activator. CF fly ash consists of CaO_3_ (48.5%), SiO_2_ (21.1%), Al_2_O_3_ (13.8%), SO_3_ (10.06%), Fe_2_O_3_ (2.25%) and others (4.29%). SA fly ash consists of Al_2_O_3_ (19.7%), SiO_2_ (36.3%), Fe2O3 (28.4%) and others (15.6%). SB fly ash consists of Al_2_O_3_ (15%), SiO_2_ (25.4%), Zn (20.6%), SO_3_ (10.9%), Fe_2_O_3_ (8.78%) and others (19.32%). The mixtures of SA fly ash and SB fly ash with GGBS, respectively, were damaged in the compressive strength test during seven days of curing. However, the built up strength of the CF fly ash and GGBS mixture can only be maintained for 7–14 days, and the compressive strength achieves 70% of that of a controlled group (cement in hardening cement paste). The strength of blended CF fly ash and GGBS started to decrease after 28 days, and the phenomenon of ettrigite was investigated due to the high levels of sulfur content. The CaO content in sustainable co-firing fly ashes must be higher than a certain percentage in reacting GGBS to ensure the strength of blended cements.

## 1. Introduction

Greenhouse gas emissions affect human health by possibly changing the climate. These emissions allegedly have negative effects on the world environment. Carbon dioxide (CO_2_) is the major constituent (approximately 75%) of the family of emissions collectively called “greenhouse gas”. Electric power plants accounted for 37% of global greenhouse gas in 2011 [1]. Fossil fuel in power plants is the dominant source in greenhouse gas generation, especially for the CO_2_ portion. Coal fired power plants are the largest contributors to CO_2_ emissions. Most countries are continuing to reduce power plant pollution by using clean energy technologies of various kinds. Circulating fluidized bed (CFB) combustion is one of the newer clean coal burning technologies.

The cement industry accounts for 5% of global carbon dioxide emission and cement is the main material in the concrete industry [2,3]. Fly ash is a byproduct of a coal-fired power plant. Recycling of power plant fly ash is a CO_2_ reduction method. According to ASTM C618 [4], coal fly ash can be substituted for a portion of the cement used to obtain the desired cementitious or pozzolanic reaction when concrete is placed. This substitution has been seeing increased use in the concrete field recently. The World Steel Association in 2011 reported that the production of global iron averages about one billion tons per year. The production of slag—a waste material from steel manufacturing—is around 150–200 kg per ton of steel product [5] depending on the quality of the raw materials used. The slag presents an environmental disposal issue if not appropriately recycled or reutilized.

The types of slag which are currently being most blended with cement to form a cementitious material are basic oxygen furnace slag (BOFS) and ground granulated blast furnace slag (GGBS) [6,7]. Blast furnace slag is a glassy material with major chemical components, such as Al_2_O_3_, CaO and SiO_2_, which can generate C-A-S-H colloid. GGBS can react similarly to hydrate Portland cement I, Ca(OH)_2_ in pozzolanic reaction. So, GGBS can partially replace Portland cement I as binder [3,6]. Much research has proven that GGBS can be substituted for Portland cement I by up to 80 wt.% [3,4,5,6,7,8,9].

There are many types of coal fired boilers, such as pulverized coal (PC) boilers, stokers, bubbling bed (BB) boilers and CFB boilers, installed in the many utility stations, cogeneration plants and steam generating facilities. PC boilers can burn oil and coal. Stoker boilers can burn oil, coal, sludge, refuse derived fuel (RDF), tire derived fuel (TDF), wood, and so on. BB and CFB boilers can burn oil, coal, biomass and waste materials [10]. Fly ash is a boiler-combustion byproduct. Fly ash in PC boilers’ firing can be recognized as a renewable substance to replace some proportion of cement in concrete applications, and is classified as a recycled material, depending on the source, in Taiwan [11,12]. Co-firing fly ash from stokers or CFB or BFB boilers is classified as a non-recyclable waste material. Recycling of those co-firing fly ashes is a crucial issue to be resolved in Taiwan. Therefore, three types of co-firing fly ashes from paper mill plants have been analyzed in this paper, because they have recently been facing the problem of waste fly ash disposal. Co-firing bituminous coal and biomass will decrease the pozzolanic activity of fly ash [12]. In order to examine the feasibility of reusing this otherwise wasted fly ash to partially replace cement, the mixing of this coal-biomass fly ash with GGBS is tested.

**Figure 1:**
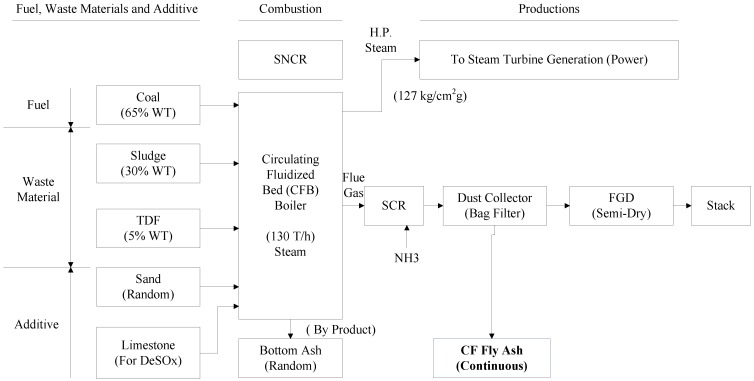
Circulating fluidized beds (CFB) combustion process.

This paper presents co-firing fly ashes produced in three boilers. Type CF fly ash is the byproduct of 130 T CFB boilers when burning bituminous coal, paper sludge and TDF (Refer to Figure 1). Paper sludge is the waste material from paper mill pulp waste treatment systems, and is a non-hazardous material. It is normally disposed by landfill or refill to kiln as raw materials. Landfill presents the risk of future leakage to the soil. It is not a positive way to dispose waste and requires special approval from local government. TDF is composed of used tires that have been shredded prior to burning. Alternate disposal of scrap tires is in stockpiles or landfill. Landfill presents a number of disposal issues, and stockpiles are a possible source of dengue or other mosquito-borne diseases. The shredded tires can be mixed with coal or other fuels burning in cement kilns, power plants or paper mills. TDF is legally allowed to be co-fired with other fuels in stokers or CFB boilers in cogeneration plants in Taiwan. Type SA fly ash comes from a 65 ton/hr. stoker boiler burning bituminous coal and TDF. Type SB fly ash is produced by a 30 ton/hr. stoker boiler burning bituminous coal and refuse derived fuel (RDF). RDF is the waste material of paper mill processing. To reuse these otherwise wasted materials (sludge, TDF and RDF), they are burned in paper mill plants in three boilers. The strength of these fly ashes is built up by pozzolanic reaction in mortar, and is better than the strength of PC fly ashes [13]. If these three fly ashes can be reused as legally recycled waste materials after special treatment or mixing with other materials, it constitutes a tremendous contribution to the paper mill industrial. This is the purpose of this paper [14,15,16].

Energy consumption for GGBS only lies in the grinding process, which generates some CO_2_ emission. So, adding 10% GGBS by weight to Portland cement I will reduce around 10% of CO_2_ emissions in the Portland cement manufacturing process [17,18]. GGBS experiences alkali reaction or pozzolanic reaction and can be a partial substitute for Portland cement as the binder. Many reports have proven that up to 80% of Portland cement can be replaced by GGBS, which may abate CO_2_ emissions also. GGBS is occasionally used as the stabilizer to increase concrete volume stabilization because GGBS reacts in the same way as pozzolanic reactions. It can improve the concrete volume expansion issue caused by free CaO [3,6,19,20]. The hydration of GGBS is slow compared with Portland cement, which demonstrates lower strength in the early stages and higher strength at later stages [21].

Consequently, to reutilize the waste of co-fired fly ashes as an additive to cement or other attractive alternatives, the laboratory investigators undertook an innovative approach to mix GGBS and co-fired fly ashes together to determine their mechanical and cementitious characteristics in blended cement. In SEM, after mixing GGBS with GGBOS with an optimal proportion of 50%:50% during 7 days and 28 days of curing, hexagonal platelets of C-A-S-H colloid were found [6]. This finding is in line with the reactions in CF5G5 (50% CF fly ash: 50% GGBS) at 28 days age whereby Ca(OH)_2_ reacted with SiO_2_ and Al_2_O_3_ of GGBS powder forming C-A-S-H. Therefore, CF fly ash could act as an alkali activator for GGBS due to its similarity to GGBOS.

## 2. Materials

An overview of the chemical composition of co-firing fly ash, GGBS, Portland cement and the blended materials are described below.

### 2.1. CF Fly Ash (Co-Firing)

CF fly ash is the byproduct of co-firing among bituminous coal, sludge from the paper mill pulp process and TDF. The total content of Al_2_O_3_ (13.8%), SiO_2_ (21.1%) and Fe_2_O_3_ (2.25%) is approximately 37.15%, which is less than the 70% required for ASTM-C593 [22] for fly ash cement. However, there is higher content of CaO (48.5%). CaO can be formed as Ca(OH)_2_ by adding water to become a stabilizer, as in GGBS. SO_3_ content is around 10.16%, which is higher than the normal minimum requirement in fly ash cement of containing less than 4% blended cement. The volume expansion is an issue to be assessed. The specific gravity and surface values are 2.75 g/cm^3^ and 3886 cm^2^/g, respectively. Therefore, CF fly ash mixed with GGBS can be assumed to have enough compressive strength according to ASTM C895-14 [23] blended cement.

### 2.2. SA Fly Ash (Co-Firing)

Type SA fly ash is the byproduct of co-firing with bituminous coal (82%) plus TDF (18%). The purpose is to dispose of waste tires as a cheap, high-energy value fuel. Type SA fly ash consists of Al_2_O_3_ (19.7%), SiO_2_ (36.3%), Fe_2_O_3_ (28.4%) and others (15.6%). The first three components occupy 84%, which is larger than 70% as specified on fly ash cement ASTM C-618 [4]. The percentage of three key elements is similar with fly ash class F [4]. MgO is less than the ASTM C-618 [4] upper limit (1.66%). One issue is CaO, which is around 2.1%. The fly ash color of coal co-firing with TDF is dark due to the black carbon retained in the fly ash. The specific gravity and surface values are 2.8 g/cm^3^ and 3116 cm^2^/g, respectively.

### 2.3. SB Fly Ash (Co-Firing) 

SB fly ash is the byproduct of co-firing by bituminous coal and RDF. Its chemical analysis is Al_2_O_3_ (15%), SiO_2_ (25.4%) and Fe_2_O_3_ (8.78%). The total of three major elements is 49.18%, which is less than fly ash class F [4] requirement of 70% and fly ash class C of 50%. There is high SO_3_ content (10.9%). Very special metal contents of Cl (4.70%) and Zn (20.6%) were found because RDF was produced by waste industrial paper, which contained Cl and Zn. The specific gravity and surface values are 2.35 g/cm^3^ and 3608 cm^2^/g, respectively. The chemical composition is similar to standard class C fly ash.

### 2.4. Grounded Granulated Blast-Furnace Slag (GGBS)

GGBS is the byproduct of iron blast furnace smelting. GGBS powder has a specific surface area of 5892 m^2^/kg with a specific gravity of around 2.89. Its major chemical constituents are CaO (41.16%), SiO_2_ (33.42%), Al_2_O_3_ (13.35%) and MgO (7.76%). Total content of SiO_2_, Al_2_O_3_ and Fe_2_O_3_ is 46.70% in which those compounds meet the requirements of ASTM C595/C595M-13 [24]. Referring to referenced technical reports [3], it is suggested to mix GGBS and GGBOS together in developing innovative new materials for industrial cement and to reduce CO_2_ emissions as well. The aforementioned two waste materials can be mixed to develop the normal cementitious material substituting cement in industrial concrete [3]. It is also indicated in the test results that increasing the weight percentage of GGBS can increase the free CaO or Ca(OH)_2_ concentration that creates the alkaline environment to stimulate the pozzolanic reaction with Al_2_O_3_ and SiO_2_ in GGBS, which generates compressive strength and strengthened microstructure [3]. Referring to the papers, the free CaO and free MgO react with water to form Ca(OH)_2_ and Mg(OH)_2_, which results in volumetric expansion [3,6,7,9]. So, GGBS can be deemed as a stabilizer, the same as PC fly ash, which is normally added to stabilize converted slag [3,5,12,13]. GGBS normally remains in alkali environments producing strength. Putting GGBS in water causes the CaO to form (GGBS already requires 41.16% of CaO to react with H_2_O to form Ca(OH)_2_). Consequently, mixing GGBS with cement can generate a pozzolanic or alkali-activated reaction.

According to Tsai’s paper on the mechanical and cementitious characteristics of GGBS and GGBOF blended mortar, the compressive strength could reach 80%–90% of ordinary Portland cement I [3,5]. The concept and finding of the referenced papers may be applied to reuse the waste material of CFB boiler fly ash in the co-firing process (burning coal and wasted materials). The characteristics of CFB or stoker co-firing fly ash are dependent on the fuels or additives. Theoretically, it is different from PC (pulverized coal) boiler fly ash, which burns pure coal only. Fly ash produce in co-firing processes in either CFB boilers or stoker boilers should not partially replace cement in concrete production in accordance with current regulations in Taiwan. Special permission is required to utilize co-firing fly ash to produce blended cement. So, this paper discusses the feasibility of an optimal mixing ratio for co-firing fly ash mixes with GGBS as blended cement according to ASTM C595/C595M-13 [24]. The mixing of each CF, SA and SB co-firing fly ash with GGBS to determine compressive strength characteristics is experimentally studied.

## 3. Experimental Section

### 3.1. Blended Materials

GGBS was mixed with three types of fly ash individually in various proportions substituting Portland cement as a binder. Compressive strength experiments for the following blended materials were conducted. There was (1) fly ash CF + GGBS; (2) fly ash SA + GGBS; (3) fly ash SB + GGBs; and (4) 100% cement, as shown in Table 1. This is to determinate the hydration of the four blends.

Electron microscope was used to verify the particle shape of the three types of fly ashes and cements. Compressive strength testing, X-ray diffraction (XRD) and scanning electron microscopy (SEM) were used to verify the hydration.

**Table 1:** Mixture proportions of blended materials (%).

Proportion No.	CF	SA	SB	C	GGBS
Cement	-	-	-	100	0
CF5G5	50	-	-	-	50
SA5G5	-	50	-	-	50
SB5G5	-	-	50	-	50

**Figure 2:**
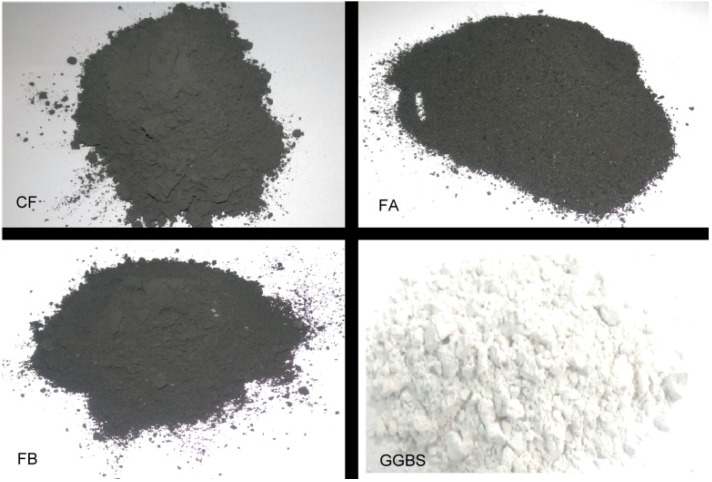
Images of three co-firing fly ashes and ground granulated blast-furnace slag (GGBS).

This study aimed to examine the optimal mixing ratio between each co-firing fly ash and GGBS. This presents some benefits the environment and business sectors. One benefit is re-utilizing the sustainable materials of co-firing fly ashes, because the owner of a paper mill plant pays for disposal of those waste materials. Another is that if those otherwise wasted fly ashes can be used as pozzolanic materials with GGBS for replacing cement in concrete production projects, it can reduce cement consumption in concrete processing with reductions in CO_2_ emissions. The ground-granulated blast furnace slag is used as binder. The main variable in the experiments is to find the possible alkali activators in three fly ashes: CF, SA and SB. In Figure 2, the color of fly ash CF is dark grey. The color of fly ash SA is dark, close to black, due to the black carbon in ash. Fly ash SB’s color is between blue and black. GGBS’s color is near white. Mixture proportion of GGBS and three fly ashes is 1:1 by weight, with the water to binder ratio 0.5 for all test specimens. The characteristics of all test specimens are introduced in Table 2.

**Table 2:** Chemical composition and physical analysis of test specimens.

Chemical Composition (wt. %)	CF	SA	SB	C	GGBS (*)	GGBOS (*)
Cl	NA	NA	4.07	NA	NA	NA
Zn	NA	NA	20.6	NA	NA	NA
Mn	NA	NA	NA	NA	0	2.39
MgO	2.29	1.66	1.65	2.52	7.76	7.26
Al_2_O_3_	13.8	19.7	15	5.46	13.35	0.76
SiO_2_	21.1	36.3	25.4	21.04	33.42	12.2
SO_3_	10.06	3.49	10.9	NA	NA	0.18
K_2_O	0.24	1.24	2.47	NA	NA	NA
CaO	48.5	2.1	6.39	63.56	41.16	40.4
Fe_2_O_3_	2.25	28.4	8.78	2.98	0.21	30.2
P_2_O_5_	0.306	0.97	2.07	NA	NA	NA
TiO_2_	0.731	1.03	1.39	NA	NA	NA
Others	0.723	5.11	1.28	4.44	4.1	6.61
Physical Properties						
Specific gravity (g/cm^3^)	2.75	2.8	2.35	3.15	2.89	3.59
Specific surface (cm^2^/g)	3886	3116	3608	3713	5892	12315

* data referred to in the previous reports [3,5].

### 3.2. Compressive Strength Test

The compressive strength tests of the specimens were conducted according to ASTM C109 [25]. For each mixture, 50 mm × 50 mm × 50 mm cubes were prepared and three specimens of each mixture were tested at the age of 7, 14 and 28 days to investigate the average compressive strength.

### 3.3. Microstructure Analysis

In SEM analysis, three specimens for each blended materials with dimensions of 10 mm × 10 mm × 3 mm were conducted. There were a total of 12 test specimens. Energy dispersive spectroscopy is also equipped with SEM. The specimens were analyzed by XRD also. The specimens were stored in powder, and XRD was used with a Cu-Kα radiation at room temperature, which were scanned at 2θ = 10° − 80°.

## 4. Results and Discussion

### 4.1. Compressive Strength of Co-Firing Fly Ashes, Cement and GGBS/GGBOS Paste

**Figure 3:**
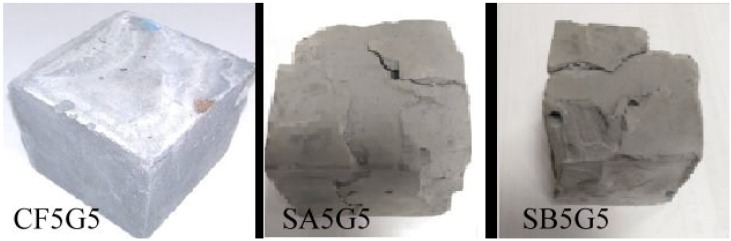
Images of CF5G5, SA5G5 and SB5G5 after compressive test. (CF5G5: 50% fly ash CF + 50% GGBS, SA5G5: 50% fly ash SA + 50% GGBS, SB5G5: 50% fly ash SB + 50% GGBS)

**Figure 4:**
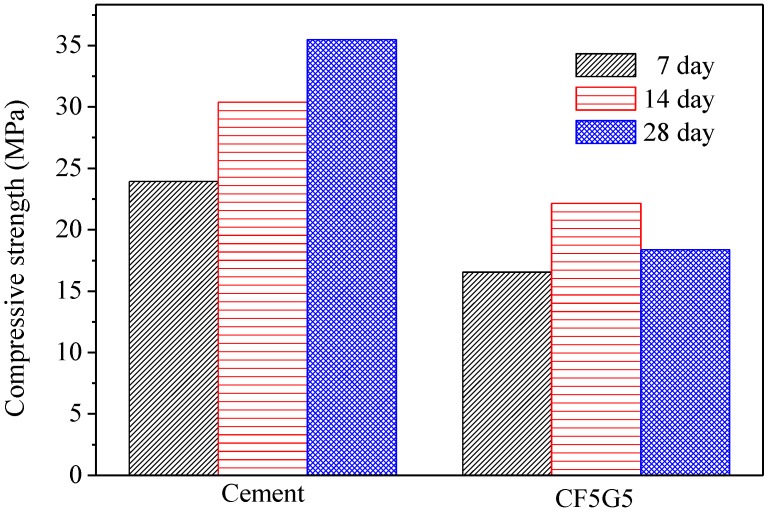
Compressive strength of co-firing fly ash and GGBS blended cement mortar. (GGBS: ground granulated blast-furnace slag, CF5G5: 50% fly ash CF + 50% GGBS)

**Figure 5:**
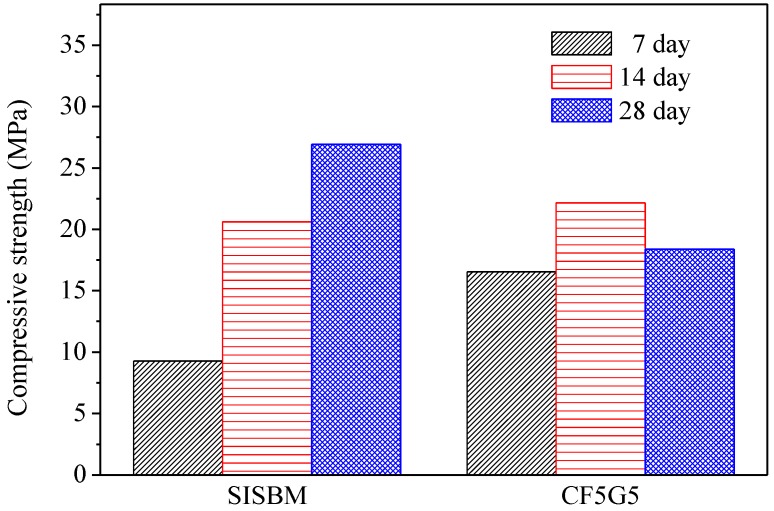
Compressive strength of SISBM and CF5G5 blended cement mortar. (SISBM: steel and iron slag blended material, CF5G5: 50% fly ash CF + 50% GGBS)

Table 3 presents the compressive strength of five test specimens including CF5G5 (50% CF + 50% GGBS), SA5G5 (50% SA + 50% GGBS), SB5G5 (50% SB + 50% GGBS), C (100% ordinary Portland cement I) and SISGM (mixture of GGBS and GGBOS). The control specimen is 100% ordinal Portland cement. The test results show only mixing of fly ash CF and GGBS with mixture proportion of CF5G5 generates compressive strength. The strength of CF5G5 was 16.55 MPa at 7 days and increased to 22.16 MPa at 14 days and went down to 18.39 MPa at 28 days. The mixing of SA5G5 and SB5G5 essentially did not lead to a reaction and were damaged by compressive test in seven days, as shown in Figure 3. For SISGM data, refer to Tsai’s paper S4I6 of Table 3 [3]. With the test results shown in Table 3, a control group cement paste, the ratio of CF5G5 and the referenced test results of Tsai’s paper are integrated in Figure 4 and Figure 5. Figure 4 shows that CF5G5 during day 7–14, compressive strength gradually increased by increasing curing time. However, a downward trend appeared at 28 days, in which the peak point of intensity can be determined as being 70% of the control group’s compressive strength. This result shows that there was only the mixture of CF5G5 reacted with water to produce compressive strength among the three combinations (CF5G5, SA5G5 and SB5G5). It is indicated that only CF fly ash can effectively stimulate the potential characteristics of GGBS and generate potential strength, but its intensity of growth can only be maintained at the early stage. From 28 days, compressive strength experiences a downward trend. According to the specification ASTM C595/C595M-13 [24], composition of SO_3_ content in the blended cement materials is limited to not exceeding 4%, as when SO_3_ content is too high to cause reaction with certain components of the mortar. This results in a hard solid volume expansion caused by the deterioration or lowering of the compressive strength. Sulfur trioxide in CF fly ash is approximately 10.6 %wt.. Converting the SO_3_ content in proportion to CF5G5 corresponds to 5.3% of total weight, which exceed the specified 4% maximum content. The high level of SO_3_ content explains why the compressive strength reduces at the late stage [26,27].

**Table 3:** Compressive strength results, MPa.

Age No.	CF5G5	SA5G5	SB5G5	Cement	SISBM (*)
7 day	17.18	NA	NA	23.14	NA
	17.5	NA	NA	24.42	NA
	14.98	NA	NA	24.21	NA
Average	16.55	NA	NA	23.92	9.27
Std.-Dev.	1.120	-	-	0.561	-
14 day	21.37	NA	NA	28.73	NA
	23.01	NA	NA	31.69	NA
	22.09	NA	NA	30.75	NA
Average	22.16	NA	NA	30.39	20.61
Std.-Dev.	0.671	-	-	1.235	-
28 day	20.91	NA	NA	35.97	NA
	16.63	NA	NA	35.18	NA
	17.63	NA	NA	35.31	NA
Average	18.39	NA	NA	35.49	26.92
Std.-Dev.	1.828	-	-	0.346	-

* SISBBM refers to Table 3, S4I6 of Tsai’s paper [3].

Figure 3 shows an image of three specimens, which are SA, SB and CF, respectively. The specimens of SA5G5 and SB5G5 were damaged during compressive strength tests. The results show that the mixture of 50% SA (wt.) + 50% GGBS (wt.) and 50% SB (wt.) + 50% GGBS (wt.) was not workable. The specimen of CF5G5 could produce strength and was feasible in carrying out further tests.

### 4.2. Impact of Strength Developing by CaO

Figure 5 shows the test results for compressive strength of CF5G5 and SISGM—which were integrated in Tasi’s paper [3,6] in using mixture no. S4I6 of GGBS and GGBOS—and found that an optimal mixture of SISGM could be used for alkali activation similar to alkaline cement, ensuring concrete hydration and generating a certain strength. According to Tsai’s paper, GGBS experienced gelation effects with a slow reaction and obtained some f-CaO in the aforementioned materials. A f-CaO reacted with water to form Ca(OH)_2_. Ca(OH)_2_ reacted with SiO_2_ and Al_2_O_3_ of GGBS powder forming C-A-S-H ingredients to produce alkali-activated or pozzolanic reactions, increasing the strength of the mixture [3,6].

**Figure 6:**
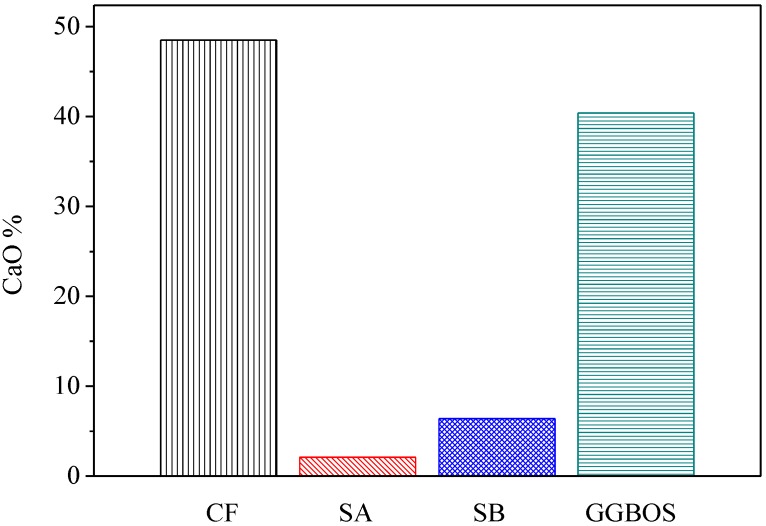
CaO contenting in CF, SA, SB and GGBOS (wt.%). (CF: fly ash of 130 ton/hr. boiler co-firing, SA: fly ash of 65 ton/hr. boiler co-firing, SB: fly ash of 30 ton/hr. boiler co-firing, GGBOS: ground granulated basic oxygen-furnace slag)

Figure 6 presents the percentage of calcium oxide content in three co-firing fly ashes in comparison with GGBOS. It is found that CaO content in CF is 48.5%, in SA is 2.1% and in SB is 6.38%. While the three fly ashes contain CaO, CF contains significantly more than the other two. The test results of compression found the CF5G5 mixture was only able to produce strength, which supports Tsai’s research [3,6]. However, the SA5G5 and SB5G5 mixtures also contained a small amount of CaO. It did also foresee that insufficient CaO content in mixing mortar could not effectively stimulate GGBS’ potential activation. It meant that CaO can effectively stimulate the GGBS powder to live up to its potential strength, but the content must be at a minimum level to be alkali-activated. Figure 6 shows that GGBOS contained 40.4% and CF5G5 contained 48.5% of CaO [3,6]. Consequently, in investigating the recycling of sustainable materials of co-firing fly ashes as an alkali activator with GGBS blended cements, CaO content should represent more than 40% of total composition, in order to stimulate GGBS powder to produce strength.

### 4.3. Microstructures

**Figure 7:**
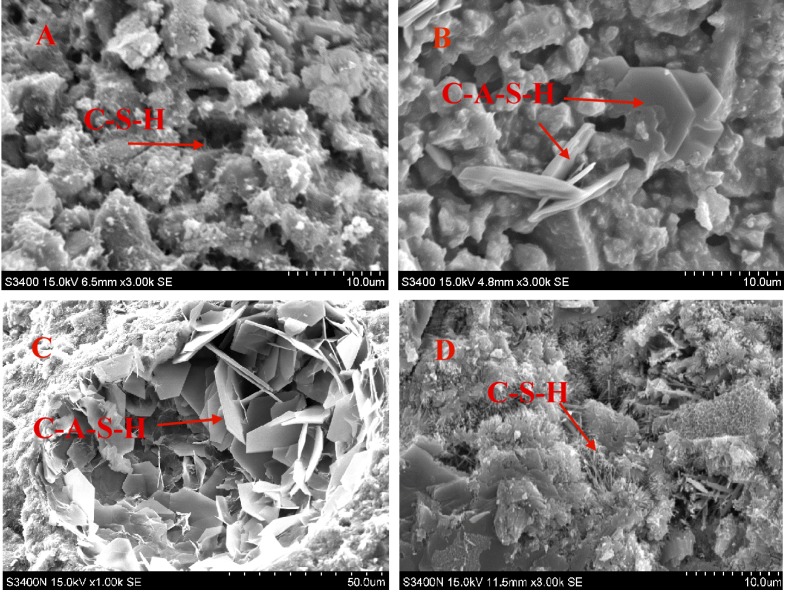
SEM micrographs of mixture CF co-firing fly ash and GGBS (28 days) (Images of sub-figure A&B are the surface structure of CF5G5. Images of sub-figure C and D referred to Figure 8 of Tsai’s paper [3]. CF: fly ash of 130 ton/hr. boiler, GGBS: ground granulated blast-furnace slag. C-S-H and C-A-S-H refers to Table 4).

The microstructure of CF co-firing fly ash mixed with GGBS is shown in Figure 7. The SEM analysis used the magnified 3000 times image of CF5G5 mixture and observations found that the surface structure of CF5G5 is mainly sharp needles and hexagonal flakes, as shown in Figure 7A,B. The EDS analysis showed the major elements are Ca, Si with O and Ca, Si, Al with O, which, thus, infers those elements are a colloid of C-S-H and C-A-S-H. Based on previous study [3], C-S-H and C-A-S-H are the key factors in producing strength in cementitious materials. This is shown in Figure 7C and Figure 7D, based on previous reports [3,6]. Thus, it was confirmed the main reason for the strength production of CF5G5 came from C-S-H and C-A-S-H.

**Figure 8:**
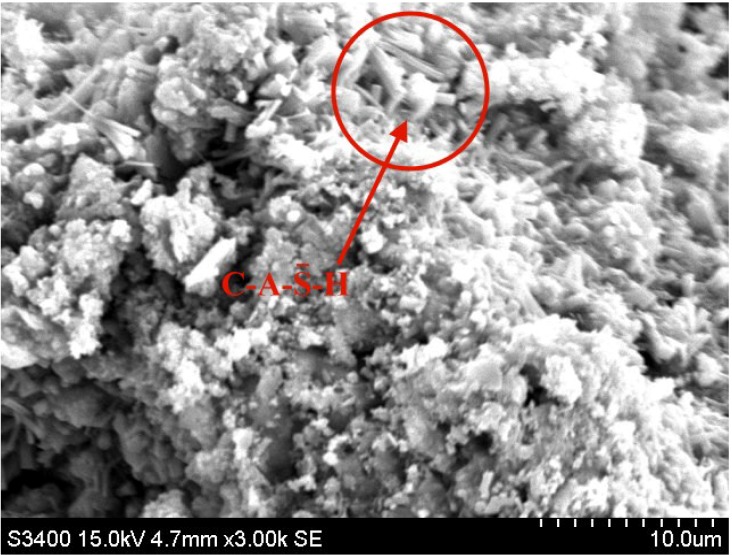
SEM micrograph of mixture CF and GGBS blended cement (28 days). (CF: fly ash CF, GGBS: ground granulated blast-furnace slag, C-A-$\overset{─}{\text{S}}$-H: C = CaO, A= Al_2_O_3_, $\overset{─}{\text{S}}$ = S, H = H_2_O)

Figure 8 also shows C-S-H and C-A-S-H colloids on the surface of CF5G5. There were many hexagonal pillars in other locations that are mainly elements of Ca, S, Al and O. C-A-H of those elements reacted with a high percentage of sulfur becoming C-A-$\overset{─}{\text{S}}$-H, or ettringite. The results proved the previous inference that the late-stage drop in strength of CF5G5 mixture is really caused by high sulfur content.

### 4.4. XRD

**Figure 9:**
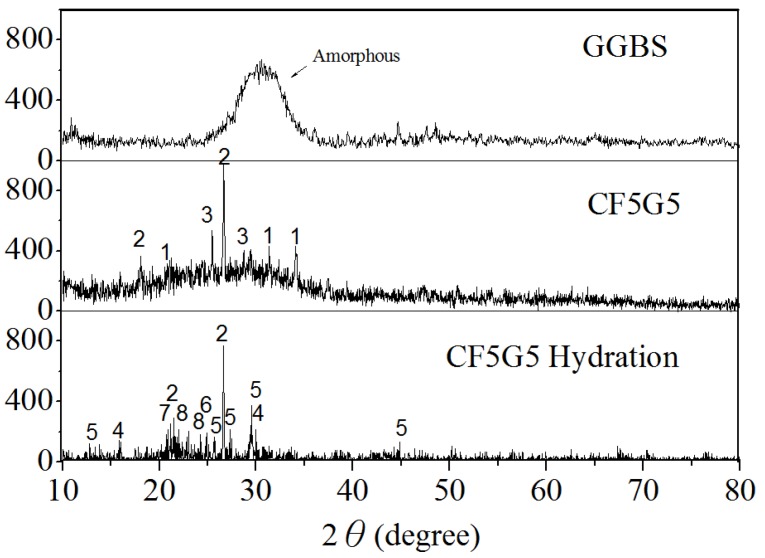
X-ray diffraction of GGBS, CF5G5 and CF5G5 hydration. (GGBS: ground granulated blast-furnace slag, CF5G5: 50% fly ash CF + 50% GGBS)

Figure 9 and Table 4 show the X-ray diffraction and XRD pattern for the GGBS, mixture ratio of CF5G5 and phase structure of CF5G5 powder, and highlight that GGBS is an amorphous structure. After mixing GGBS and CF fly ash, the crystalline phase will appear by CaO, SiO_2_ and CaSO_4_. After hydration of CF5G5, the phases of SiO_2_, C-S-H, C-A-S-H, CaSO_4_, CaSO_4_ (H_2_O)_8_, C-A-$\overset{─}{\text{S}}$-H, and Al_2_H_18_O_24_S_4_ appeared, whereby C-S-H and C-A-S-H are the main sources of concrete strength. The elements Ca$\dot{\text{S}}$O_4_, CaSO_4_ (H_2_O)_8_, C-A-$\overset{─}{\text{S}}$-H, and Al_2_H_18_O_24_S_4_ have S-containing mixture, which may lead to the expansion of the volume of concrete, which may cause deterioration. This result supports previous assumptions that the generation of hydration by mixing GGBS powder with co-firing fly ash is feasible and achievable. The main reason for increased strength when mixing CF5G5 hardened paste is the presence of C-S-H and C-A-$\overset{─}{\text{S}}$-H. Evidence of similar phases has been demonstrated in work by Dung [28]. It was also learned that the strength of CF5G5 gradually decreased after curing for 28 days, in which the main reason may be too much sulfur content, resulting in specimen deterioration.

**Table 4:** XRD patterns of the mixture CF co-firing fly ash and GGBS. (CF: fly ash CF, GGBS: ground granulated blast-furnace slag)

No.	1	2	3	4	5	6	7	8
**Mixtures**	CaO	SiO_2_	CaSO_4_	*C-S-H	*C-A-S-H	CaSO_4_ (H_2_O)_8_	*C-A-$\overset{─}{\text{S}}$-H	Al_2_H_18_O_24_S_4_

Notes: * Abbreviation; CaO(C), Al_2_O_3_(A), SiO(S), H_2_O(H) and S($\overset{─}{\text{S}}$).

## 5. Conclusions

1. CF fly ash can effectively stimulate the potential properties of GGBS powder to produce compressive strength. The strength is increased and maintained at early stages, from 7 to 14 days’ curing. Strength will start to decline after 28 days of curing. The maximum strength from mixing GGBS and CF co-firing fly ashes achieved is around 70% of that of the control group, cement hardened cement paste.
2. This study investigates whether waste fly ash can complement GGBS in providing strength, and found that the CaO content needs to be above a certain level for it to act as an alkali activator and generate strength.
3. The analysis of chemical composition and SEM micrographs shows that the strength of CF5G5 was reduced after 28 days. The main reason may be the higher content of sulfur resulting in the formation of ettringite. This phenomenon is caused by the binder volume expansion.
